# Comparison of statin plus ezetimibe with double-dose statin on lipid profiles and inflammation markers

**DOI:** 10.1186/s12944-018-0909-z

**Published:** 2018-11-23

**Authors:** Na-Qiong Wu, Yuan-Lin Guo, Cheng-Gang Zhu, Ying Gao, Xi Zhao, Di Sun, Jing Sun, Rui-Xia Xu, Geng Liu, Qian Dong, Jian-Jun Li

**Affiliations:** Division of Dyslipidemia, State Key Laboratory of Cardiovascular Disease, Fu Wai Hospital, National Center for Cardiovascular Disease, Chinese Academy of Medical Sciences and Peking Union Medical College, No 167 BeiLiShi Road, XiCheng District, Beijing, 100037 China

**Keywords:** Statin, Ezetimibe, Combination, LDL-C, Ox-LDL, Chinese

## Abstract

**Background:**

Achievement of low-density lipoprotein cholesterol (LDL-C) goal is the most important for the patients with atherosclerotic cardiovascular diseases (ASCVD) who received lipid-lowering therapy. It is unclear that whether combination of ezetimibe with statin is superior to double-dose of statin regarding both of the lipid-lowering efficacy and improvement of inflammation in Chinese patients with ASCVD. Therefore, this study was performed to compare the effects of these two regimes on lipid profiles and inflammation markers.

**Methods:**

In this randomized control study, ninety eight patients with ASCVD, who were naïve to statins or other lipid-lowering agents, were enrolled into the study, and randomly assigned into two groups, A40 group (atorvastatin 40 mg/d, *n* = 50), A20E10 group (atorvastatin 20 mg/d combined with ezetimibe 10 mg/d, *n* = 48).The patients were followed up at week 4 and week 12 after treatment. The lipid profiles and oxidative low-density lipoprotein cholesterol (ox-LDL) were measured at the end of study.

**Results:**

There were no differences in clinical characteristics including lipid, ox-LDL and hypersensitive C reactive protein (Hs-CRP) among groups at baseline. However, the average level of LDL-C was lower in group A20E10 than that in group A40 significantly (1.59 ± 0.44 mmol/L vs 1.99 ± 0.56 mmol/L, *p* = 0.001) during follow-up at week 12 after treatment. Importantly, the higher rate of achievement of LDL-C goal was attained at group of combination statin with ezetimibe (79.2% in group A20E10 vs 50.0% in group A40, *p* = 0.016). The difference of the level of ox-LDL between both the groups after 12 weeks treatment had not statistical significance (3.63 ± 1.13 U/L in group A20E10 vs 4.14 ± 1.32 U/L in group A40, *p* = 0.077).Similarly, the level of Hs-CRP between both the groups after treatment was not significantly different (*p* > 0.05).

**Conclusions:**

In this randomized study, the data showed that a combination of moderate statin and ezetimibe achieved more reduction of LDL-C compared to the double-dose statin but similar impact on inflammation markers.

## Introduction

According to the 2013 American Cardiology College/American Heart Association (ACC/AHA) guideline of cholesterol management, the patients at very high risk of atherosclerotic cardiovascular diseases (ASCVD) should take high intensity statins whatever the low-density lipoprotein cholesterol (LDL-C) level when initiation statin treatment [1], however, evidences showed that there was racial difference in statin tolerance and safety problems between Chinese and Caucasians [2]. High intensity statins were defined as atorvastatin 40-80 mg/d or rosuvastatin 20-40 mg/d in 2013 ACC/AHA guideline. Unfortunately, in China, rosuvastatin 40 mg/d is prohibited to prescribe for patients due to no approval from Chinese State Food and Drug Administration (SFDA). In addition, lack of evidence about benefit and safety of atorvastatin 80 mg/d on Chinese patients supported Chinese doctors to prescribe it. Hence, the Chinese adult lipid management guideline (2016 revision edition) do not recommend high-dose statin such as atorvastatin 80 mg/d or rosuvastatin 40 mg/d to use in real world clinical practice [3].

Nevertheless, both 2016 European Society of Cardiology/European Atherosclerotic Society (ESC/EAS) guideline and 2016 Chinese guideline of cholesterol management recommended that for the patients at very high risk of ASCVD, the goal of LDL-C should be < 1.8 mmol/L or a reduction of at least 50% if the baseline level of LDL-C was 1.8–3.5 mmol/L [3, 4]. Moreover, IMPROVE-IT trial (Improved Reduction of Outcomes: Vytorin Efficacy International Trial) demonstrated that combination ezetimibe with simvastatin can reduce the level of LDL-C more when compared with simvastatin monotherapy in the patients with acute coronary syndrome and reduce the rate of major adverse cardiovascular events [5], which might suggest that combination ezetimibe with moderate statin may achieve more LDL-C reduction in Chinese high risk patients compared to double-dose statin.

Oxidative stress plays an important role in atherosclerosis and oxidative low-density lipoprotein cholesterol (ox-LDL) has been demonstrated to be a principle lipid-related marker for predicting the severity and outcomes in patients with ASCVD besides LDL-C and non-high-density lipoprotein cholesterol (non-HDL-C) [6]. Additionally, A very large number of studies have indicated that hypersensitive C-reactive protein (Hs-CRP) was a useful inflammatory marker for judging the prognostic outcomes in a variety of cardiovascular diseases [7, 8]. Interestingly, previous studies showed a powerful impact of statins on oxidative and inflammatory response such oxLDL and Hs-CRP. Based on these evidences and situation in China, we designed this study to compare combination of statin plus ezetimibe and double-dose statin regard to LDL-C reduction and inflammation markers improvement for the Chinese patients at very high risk of ASCVD.

## Methods

### Study design and patient population

The study protocol was reviewed and approved by the Ethics Committee of FuWai Hospital, and informed consent was obtained from all patients. In our randomized, open-label, prospective study, 98 patients naïve to statins or other lipid-lowering agents were enrolled who were diagnosed as ASCVD including coronary artery disease (CAD) or carotid artery disease. The inclusion criteria was as following, 1) the patients with stable angina received coronary angiography showing at least one coronary artery (left anterior descending artery, left circumflex artery, or right coronary artery) stenosis ≥50%, or 2) the patient received carotid ultrasound showing at least one carotid artery stenosis ≥50%, 3) no history of statin treatment or use of other drugs known to affect blood lipids within 3 months, 4) age 18–70. Patients with triglycerides (TG) ≥500 mg/dL (5.6 mmol/L), previous acute coronary syndrome within 1 month, serious heart failure or arrhythmia, infectious disease within 1 month, serious liver or renal dysfunction, autoimmune disease, malignant tumor, pregnancy or lactation, or psychiatric disorders were excluded from the study. In addition, patients with one of the following laboratory values above 3 times the upper limit of normal laboratory range: serum alanine aminotransferase (ALT), serum aspartate transaminase (AST), and creatine phosphokinase above 5 times the upper limit of normal were also excluded.

### Clinical and laboratory assessments

Venous blood samples were obtained in the morning after 12 h fasting and were blindly assessed with regard to treatment allocation. All laboratory measurements were performed at the laboratory of Biochemistry of FuWai Hospital. Concentrations of total cholesterol (TC), triglycerides (TG) and high-density lipoprotein cholesterol (HDL-C), Apolipoprotein (Apo) A1 and ApoB, as well as lipoprotein a [Lp(a)] were measured using an automatic biochemistry analyzer (Hitachi 7150, Tokyo, Japan).TC, TG, HDL-C levels were measured using an enzymatic assay. LDL-C was calculated with the Friedewald formula. Apo A1, ApoB, and Lp(a) levels were measured using a turbidimetric immunoassay. Serum creatinine, liver and muscle enzymes were measured using conventional methods. Plasma levels of ox-LDL were measured by a competitive-enzyme-linked immunosorbent assay using a specific murine monoclonal antibody (4E6) according to the instructions provided by the manufacturer.

### Statistical analysis

Data analyses were performed using SPSS 19.0 statistical software. Measurement data are expressed as the means ± standard deviations (SD); Enumeration data are expressed as absolute frequencies and percentages. For between-group comparisons of basic demographic characteristics and clinical laboratory indicators, analysis of variance (ANOVA) was used for normally distributed measurement data. The paired t test was used to compare the baseline and post-treatment at week 4 and week 12 in each group. Independent-samples t test was used for comparison between two treatment groups. The chi-square analysis was used to examine the categorical data between treatment groups. All of the tests were conducted at a level of significance of *p* = 0.05.

## Results

### Baseline characteristics

Ninety-eight patients with ASCVD were enrolled and assigned randomly into two groups, including A40 group (A40, *n* = 50) and A20E10 group (A20E10, *n* = 48). The baseline characteristics including age, sex had no significant difference between both the groups (all *p* > 0.05).Similarly, no significant difference of the average baseline level of TC, TG, HDL-C, LDL-C, Lp(a), ApoB ApoA1, and ox-LDL level was found in both the groups (all *p* > 0.05). The baseline hepatic and renal function was normal and there was no significant difference in the two groups (all *p* > 0.05). The difference of the baseline fasting blood glucose level and HsCRP had no significance in both the groups (all *p* > 0.05, Table 1).

**Table 1:** Baseline characteristics of the enrolled patients in each group

	A20E10 (*n* = 48)	A40 (*n* = 50)	*P* value
Age(yrs)	56 ± 11	57 ± 8	0.6014
Male/Female	35/13	36/14	0.8207
TC(mmol/L)	4.91 ± 0.95	4.82 ± 0.73	0.645
TG(mmol/L)	1.89 ± 0.73	1.74 ± 0.79	0.332
HDL-C(mmol/L)	1.07 ± 0.25	1.14 ± 0.31	0.195
LDL-C(mmol/L)	3.31 ± 0.89	3.20 ± 0.90	0.540
Non-HDL-C(mmol/L)	3.91 ± 0.91	3.56 ± 0.78	0.077
Lp(a) (mg/L)	222 ± 215	160 ± 195	0.140
ApoB(mmol/L)	1.09 ± 0.29	1.10 ± 0.26	0.774
Apa A1(mmol/L)	1.30 ± 0.33	1.45 ± 0.45	0.063
ALT(U/L)	23.44 ± 15.36	19.90 ± 10.84	0.190
AST(U/L)	19.89 ± 5.99	17.80 ± 6.02	0.089
TBil(umol/L)	14.26 ± 4.19	13.96 ± 4.28	0.722
DBil(umol/L)	2.30 ± 0.64	2.34 ± 0.71	0.748
Cr(umol/L)	77.69 ± 15.57	73.36 ± 14.79	0.163
BUN(mmol/L)	5.05 ± 1.07	5.28 ± 1.16	0.327
CK(U/L)	82.13 ± 35.11	107.60 ± 117.44	0.157
FBG(mmol/L)	5.34 ± 1.00	5.76 ± 1.24	0.075
HsCRP(mg/dL)	1.74 ± 1.97	2.45 ± 3.00	0.178
ox-LDL(U/L)	6.04 ± 2.00	6.04 ± 2.00	0.998

Notes: *TC* Total cholesterol, *TG* Triglyceride, *HDL-C* High-density lipoprotein cholesterol, *LDL-C* Low-density lipoprotein cholesterol, *Lp(a)* Lipoprotein a, *ApoB* Apolipoprotein B, *ApoAI* ApolipoproteinAI, *ALT* Alanine aminotransferase, *AST* Aspartate Transaminase, *TBil* Total Bilirubin, *DBil* Direct Bilirubin, *Cr* Creatinine, *CK* Creatine Kinase, *UA* Uric Acid, *FBG* Fasting Blood Glucose, *HsCRP* High sensitive C- Reactive Protein, *ox-LDL* oxidation low-density lipoprotein

A20E10 indicates the patients who received Atorvastatin 20 mg qn plus Ezetimibe 10 mg qd,A40 indicates the patients who received Atorvastatin 40 mg qn

### Levels of lipid profile and ox-LDL

During the follow-up period at week 4 after treatment, the average level of TC was 3.13 ± 0.97 mmol/L (group A20E10), 3.50 ± 0.79 mmol/L (group A40), respectively (*p* = 0.028). The average level of LDL-C was 1.68 ± 0.74moll/L (group A20E10), 1.89 ± 0.63moll/L (group A40), respectively (*p* = 0.143).The reduction of average level of TG was 32% in group A20E10 and 20% in group A40, respectively (*p* > 0.05). The change of HDL-C level had no significant difference compared with that of baseline in the two groups (*p* > 0.05). The reduction of Non-HDL-C level after treatment was significant when compared with that of baseline in both the groups (1.89 ± 0.88 mmol/L vs 3.91 ± 0.91 mmol/L in group A20E10, 2.22 ± 0.86 mmol/L vs 3.56 ± 0.78 mmol/L in group A40, *p* < 0.001 respectively).

**Fig. 1:**
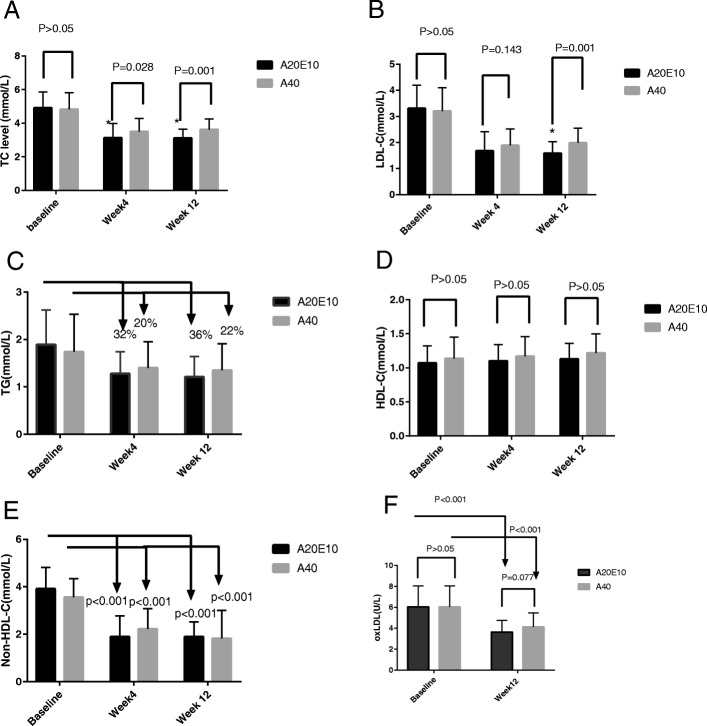
The change of serum lipid profile levels and ox-LDL in both groups during follow-up. The patients with ASCVD divided into two groups, the combination group who received Ezetimibe10mg/d with atorvastatin 20 mg/d(group A20E10, *n* = 48)and statin alone group who received atorvastatin40mg/d(group A40, *n* = 50).Serum levels of lipid profile were measured at baseline, week 4 and week 12 after treatment. **a** average TC levels,*Indicate the level of TC in group A20E10 was lower than that in group A40 significantly at week 4 and week 12 after treatment. **b** average LDL-C level,*Indicate the level of LDL-C in group A20E10 was lower than that in group A40 significantly at week 12 after treatment. **c** average TG level. **d** average HDL-C level. **e** average Non-HDL-C level. **f** average ox-LDL level. Notes: A20E10 indicates the patients who received Atorvastatin 20 mg qn plus Ezetimibe 10 mg qd,A40 indicates the patients who received Atorvastatin 40 mg qn

During the follow-up period at week 12 after treatment, the average level of TC was 3.11 ± 0.54 mmol/L (group A20E10), 3.62 ± 0.63 mmol/L (group A40), (*p* = 0.001) respectively. The average level of LDL-C was lower in group A20E10 than that in group A40 significantly (1.59 ± 0.44 mmol/L vs 1.99 ± 0.56 mmol/L, *p* = 0.001). However, the percentage reduction of average level of TG was 36% in group A20E10, 22% in group A40, respectively (*p* > 0.05), and the change of HDL-C level had no significant difference in the two groups (*p* > 0.05). The reduction of Non-HDL-C level after treatment was significant when compared with that of baseline in both the groups (1.89 ± 0.63 mmol/L vs 3.91 ± 0.91 mmol/L in group A20E10, 1.82 ± 1.18 mmol/L vs 3.56 ± 0.78 mmol/L in group A40, *p* < 0.001 respectively, Fig. 1a-e). In addition, although ox-LDL level of each group was reduced significantly during follow-up at week 12 when compared with that at baseline (3.63 ± 1.13 U/L vs. 6.04 ± 2.00 U/L in group A20E10, 4.14 ± 1.32 U/L vs 6.04 ± 2.00 U/L in group A40, *p* < 0.001, respectively), no significant difference of ox-LDL level after treatment for 12 weeks was found between group A20E10 and group A40 (*p* = 0.077, Fig. 1f).

**Fig. 2:**
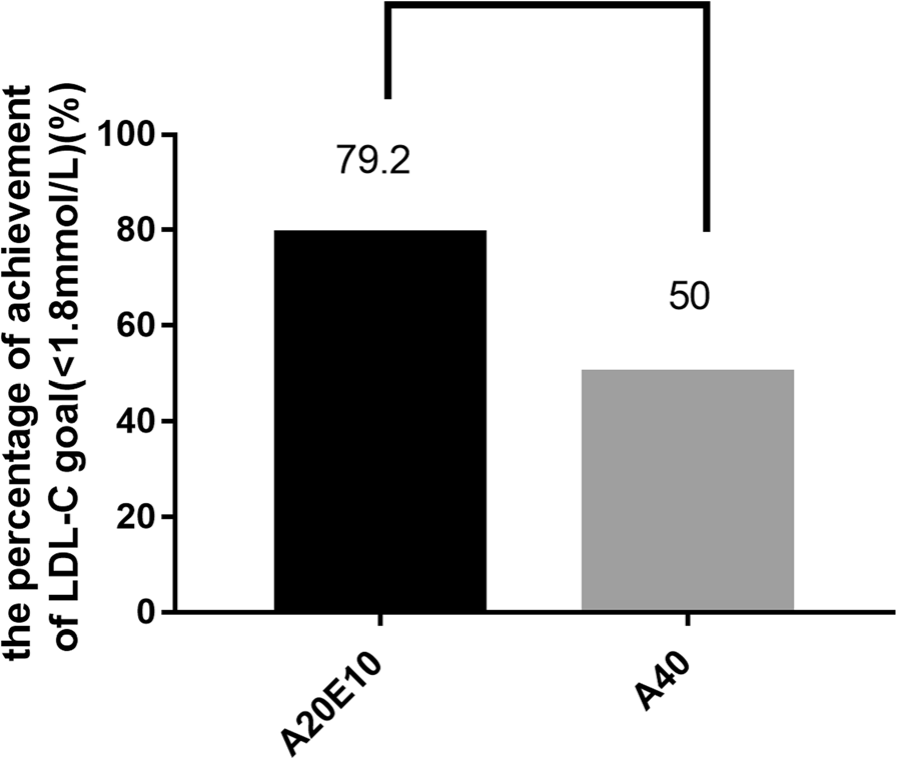
The comparison of the pecentage of achievement of LDL-C goal(< 1.8 mmol/L) between the group A20E10 and the group A40. Notes: A20E10 indicates the patients who received Atorvastatin 20 mg qn plus Ezetimibe 10 mg qd A40 indicates the patients who received Atorvastatin 40 mg qn

The higher percentage of achievement of LDL-C goal (< 1.8 mmol/L) was found in group A20E10 compared with that in group A40((79.2% in group A20E10 vs 50.0% in group A40, *p* = 0.016, Fig. 2).

### Hs-CRP

**Fig. 3:**
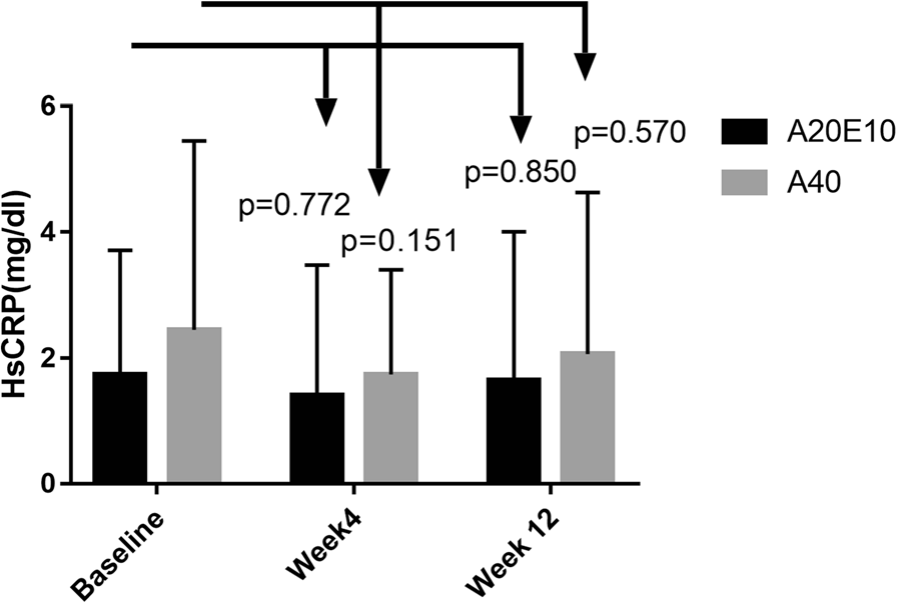
The comparison of average HsCRP level between the group A20E10 and the group A40 during follow-up. Notes: A20E10 indicates the patients who received Atorvastatin 20 mg qn plus Ezetimibe 10 mg qd A40 indicates the patients who received Atorvastatin 40 mg qn

During the follow-up period at week 4 after treatment, serum Hs-CRP level was 1.41 ± 2.07 mg/dl in group A20E10 (*p* = 0.772, when compared with that of baseline), 1.74 ± 1.66 mg/dl in group A40, (*p* = 0.151 compared with that of baseline). During the follow-up period at week 12 after treatment, serum Hs-CRP level was 1.65 ± 2.36 mg/dl in group A20E10 (*p* = 0.850, when compared with that of baseline), 2.07 ± 2.56 mg/dl in group A40, (*p* = 0.570 compared with that of baseline). The level of Hs-CRP in the two groups exhibited a tendency of reduction, even though no statistical significance was found when compared with the baseline level (Fig. 3).

### Safety evaluation

During the follow-up period at week 12 after treatment, all the patients (*n* = 98) were informed to be followed up. Self-reported side effects including fatigue or muscle pain and memory decline were recorded, and no other complains about side effects were reported. The number of the patients who complained fatigue or muscle pain was as followings, 1(2.0%) in group A40, 0 in group A20E10. The number of the patients who had ALT or AST elevation to 1–3 times of ULN was as followings, 2(4.2%) in group A20E10, 3(6.0%) in group A40. One patient (2.0%) in group A40 but nobody in group A20E10 had CK elevation 1-5times of ULN. No patients had ALT or AST elevation > 3 times of ULN or CK elevation> 5 times of ULN in both the groups.

No significant differences in ALT, AST and CK were observed in both the groups during the follow-up periods both at week 4 and week 12 after treatment (all *p* > 0.05, Table 2).

**Table 2:** Safety markers at baseline, week 4 and week 12 by group

	A20E10 (*n* = 48)	A40 (*n* = 50)	*P* value
ALT(U/L)			
Baseline	23.44 ± 15.36	19.90 ± 10.84	0.190
Week 4	30.20 ± 14.52	27.51 ± 16.78	0.410
Week 12	31.39 ± 19.38	28.39 ± 22.36	0.552
AST(U/L)			
Baseline	19.89 ± 5.99	17.80 ± 6.01	0.089
Week 4	22.84 ± 5.69	21.24 ± 6.92	0.231
Week 12	31.39 ± 19.38	28.39 ± 22.36	0.321
CK(U/L)			
Baseline	82.13 ± 35.11	107.60 ± 117.44	0.157
Week 4	95.05 ± 35.58	100.86 ± 40.46	0.466
Week 12	104.39 ± 48.86	115.60 ± 57.01	0.386

Notes: A20E10 indicates the patients who received Atorvastatin 20 mg qn plus Ezetimibe 10 mg qd,A40 indicates the patients who received Atorvastatin 40 mg qn

## Discussion

In this randomized study, we found that atorvastatin 20 mg/d plus ezetimibe 10 mg/d for 12 weeks therapy achieved more TC, LDL-C reduction for the Chinese patients with very high risk of cardiovascular diseases. Besides, the similar impact of different intensive lipid-lowering patterns on ox-LDL and Hs-CRP was found. Thereby, the study may provide additional or novel information regarding the clinical implication of statin plus ezetimibe lipid-lowering strategy.

Firstly, we investigated the efficacy of two lipid-lowering regimes in enrolled patients. As presented in Fig. 1, the lipid-lowering therapies such as atorvastatin 20 mg/d plus ezetimibe 10 mg/d and atorvastatin 40 mg/d were compared in the aspect of lipid-lowering efficacy. The lipid-lowering efficacy of both strategies was in accordance with the results reported in previous studies [9–11]. Abundant evidences demonstrated that the lower the level of LDL-C reached, the better the reduction of cardiovascular events [12–14]. During the follow-up period at week 12 after treatment, the further reduction of TC and LDL-C level was found when compared with those in the follow-up period, at week 4 after treatment, suggesting a stable lowering LDL-C effect by both moderate dose statin plus ezetimibe and double-dose statin.

Another important concern with regard to lipid-lowering therapy is safety. In the current study, we also evaluated side effects of the different lipid-lowering strategies although the sample size of the study is small. Data showed that no serious side effects happened in both groups. However, only occasional mild hepatic dysfunction or mild CK elevation was found in both the groups through more detailed observations. Thus, the combination of ezetimibe with atorvastatin had good tolerance and safety of lipid-lowering strategy.

In addition, recent reports have suggested that a synergistic effect can be obtained by concomitant administration of the cholesterol absorption inhibitor ezetimibe and a statin [15–19]. Torimoto K et al. performed the study to examine which was more effective to add ezetimibe or to increase the statin dose on LDL-C and lipoproteins in patients with type 2 diabetes who were already being treated with satins. Interestingly, they found that compared with increasing the dose of rosuvastatin, the combination of rosuvastatin and ezetimibe not only achieves quantitative but also qualitative improvement of serum lipid levels in type 2 diabetic patients, suggesting that this combination can suppress the progression of atherosclerosis [15].

Oxidation stress and inflammation represent integral features of atherogenesis and vascular diseases. It has been reported that ox-LDL is reliable marker for predicting the prognosis in ASCVD. In the present study, therefore, we investigated the change of ox-LDL level after 12 weeks lipid-lowering treatment, the level of ox-LDL was reduced significantly in both groups including combination of ezetimibe with atorvastatin (A20E10) and double-dose atorvastatin (A40). Interestingly, we found that in the two groups of our study, ox-LDL level decreased significantly and was in parallel with the reduction of LDL-C level, which is consistent with previous studies. Similar effect of ox-LDL reduction was found in both combination treatment group and double-dose statin group. Aydin MU et al. compared the effects of 80 mg daily dose of atorvastatin and 20 mg daily dose of rosuvastatin on lipid profiles and the levels of ox-LDL and inflammatory markers in ST elevation myocardial infarction (STEMI), and they found that both statin treatment regimes have comparable effects on LDL-C, ox-LDL and inflammatory markers [20]. However, in a prospective, open-label trial which enrolled 260 patients with coronary artery disease, assigned randomly into simvastatin mono-therapy group or simvastatin plus ezetimibe group, the results showed co-administration of ezetimibe with statins further lowered LDL-C levels (83 ± 23 mg/dl in S versus 67 ± 23 mg/dl in E + S; *p* < 0.0001), with significant decreases in ox-LDL level [21].

As we well known, Hs-CRP is an important inflammatory marker of ASCVD and adds prognostic information on cardiovascular risk comparable to blood pressure or cholesterol. In current U.S. guidelines, Hs-CRP carries a class IIb assessment and is most appropriate in primary prevention when clinical decisions to initiate statin therapy are uncertain. Ongoing multinational trials are pursuing whether reducing inflammation will decrease vascular event rates [22]. Further, randomized trial data addressing Hs-CRP have been central to understanding the anti-inflammatory effects of statin therapy and have consistently demonstrated on-treatment Hs-CRP levels to be as powerful a predictor of residual cardiovascular risk as on-treatment levels of LDL-C [23]. In the small sample size study with 69 patients who were allocated into simvastatin alone or simvastatin combined with ezetimibe group randomly, Krysiak R reported that the combination therapy was superior to simvastatin in influencing plasma level of Hs-CRP [24]. In the present study, the level of Hs-CRP exhibited a tendency of reduction in both the groups, when compared with the baseline level. Our data was similar to IMPROVE-IT study with regard to the changes of Hs-CRP following lipid-lowering treatment and suggested that reduction of Hs-CRP mainly depended on whether a statin is used [5].

Limitations of the current study include the open-label design, the absence of a placebo group and the relatively short duration. In addition, the small sample size is the other limitation of the study, more study is needed to confirm our findings in large sample size.

In conclusion, in this randomized study, the data showed that a combination of moderate statin and ezetimibe achieved more reduction of LDL-C compared to the double-dose statin but similar impact on inflammation markers.

## Abbreviations

ACC/AHA: American Cardiology College/American Heart Association
ALT: Alanine aminotransferase
ANOVA: Analysis of variance
Apo A1: ApolipoproteinsA1
ASCVD: Atherosclerotic cardiovascular diseases
AST: Aspartate transaminase
CAD: Coronary artery disease
ESC/EAS: European Society of Cardiology/European Atherosclerotic Society
HDL-C: high-density lipoprotein cholesterol
HsCRP: High-sensible C reactive protein
IMPROVE-IT: Improved Reduction of Outcomes: Vytorin Efficacy International
LDL-C: low-density lipoprotein-cholesterol
Lp(a): lipoprotein a
non-HDL-C: Non-high-density lipoprotein cholesterol
ox-LDL: Oxidative low-density lipoprotein cholesterol
SD: Standard deviations
SFDA: State Food and Drug Administration
STEMI: ST elevation myocardial infarction
TC: Total cholesterol
TG: Triglycerides
